# Characteristics of Women and Their Male Sex Partners Predict Bacterial Vaginosis Among a Prospective Cohort of Kenyan Women With Nonoptimal Vaginal Microbiota

**DOI:** 10.1097/OLQ.0000000000001259

**Published:** 2020-08-10

**Authors:** Supriya Dinesh Mehta, Walter Agingu, Rachel K. Nordgren, Stefan J. Green, Dulal K. Bhaumik, Robert C. Bailey, Fredrick Otieno

**Affiliations:** From the ∗ Division of Epidemiology and Biostatistics, University of Illinois at Chicago School of Public Health, Chicago, IL; † Nyanza Reproductive Health Society, Kisumu, Kenya; ‡ Research Resources Center, University of Illinois at Chicago College of Medicine, Chicago, IL

## Abstract

Up to 50% of women with nonoptimal vaginal microbial community state type (CST) have bacterial vaginosis (BV). Little is known about what distinguishes women with and without BV diagnosis within nonoptimal CST. We identified features of women and their male sex partners associated with BV among women with nonoptimal vaginal CST.

**Methods:**

In this prospective study, 252 heterosexual couples were observed at 1, 6, and 12 months after baseline. Microbiomes were characterized in cervicovaginal lavage and penile meatal swabs through high-throughput 16s ribosomal RNA gene amplicon sequencing. Nonoptimal CST was defined as CST-IV. Bacterial vaginosis was defined as a Nugent score of 7 to 10. Generalized estimating equation analysis estimated adjusted odds ratios (aORs) for BV among women with nonoptimal CST.

**Results:**

At baseline, women with nonoptimal CST were a median age of 22 years, 44% had BV, 16% had HIV, and 66% had herpes simplex virus (HSV) type 2. Male partners were a median age of 27 years, 12% had HIV, 48% had HSV-2, and 55% were circumcised. Within nonoptimal CST, *Sneathia sanguinegens*, *Prevotella* species, *Prevotella amnii*, and Clostridiales, BV-associated bacteria-2 were statistically significantly enriched in observations with BV. In multivariable generalized estimating equation controlling for CST, HIV, and HSV-2, BV was increased among women with CST-IVA (aOR, 1.91; *P* = 0.087), HIV (aOR, 2.30; *P* = 0.051), HSV-2 (aOR, 1.75; *P* = 0.065), and enrichment of male partner penile taxa: *Dialister* (aOR, 1.16; *P* = 0.034), *Megasphaera* (aOR, 1.22; *P* = 0.001), and *Brevibacterium* (aOR, 1.13; *P* = 0.019).

These results provide insights into factors differentiating women with BV among those with nonoptimal vaginal CST. Interrupting the sexual exchange of penile and vaginal taxa may be beneficial for preventing pathologic state of vaginal microbiome.

## Abstract 2

Within nonoptimal vaginal microbial community state type, bacterial composition varied by Nugent bacterial vaginosis status, and Nugent bacterial vaginosis was associated with specific penile taxa, controlling for vaginal community state type, herpes simplex virus 2, and HIV.

Supplemental digital content is available in the text.

Bacterial vaginosis (BV) is a common condition affecting up to 20% of women worldwide^1^ and 20% to 50% of women in sub-Saharan Africa.^2^ Bacterial vaginosis is associated with increased risk of HIV acquisition^3^ and other sexually transmitted infections (STIs).^4^ The cost of BV is substantial, stemming from increased risk of adverse outcomes in pregnancy, including preterm birth and premature rupture of membranes.^1,5^ Bacterial vaginosis represents a polymicrobial shift in the vaginal microbiome, often from a *Lactobacillus-*dominant community to one that is diverse, with several species of anaerobic bacteria.^6^ For women presenting with symptoms, meeting at least 3 of 4 Amsel’s criteria^7^ or having a Nugent score of 7 to 10^8^ are indications for treatment, primarily with topical or oral antibiotics.^9,10^ Despite the clinical and public health significance of BV, there are no guidelines or recommendations for screening for BV among asymptomatic women. This stems from limited evidence regarding to what extent asymptomatic BV represents a pathologic condition.

As characterized by 16s rRNA gene sequencing, quantitative polymerase chain reaction (PCR), and metaproteomics, McKinnon et al.^11^ categorize vaginal community state type (CST) IV (“depleted of lactobacilli with abundant anaerobes”) as “molecular BV,” based on its consistent association with increased risk of HIV and mucosal inflammation in an expansive review of the literature. Community state types I (*Lactobacillus crispatus* dominated), II (*Lactobacillus gasseri* dominated), III (*Lactobacillus iners* dominated), and V (*Lactobacillus jensenii* dominated) are not considered molecular BV and have not been consistently associated with adverse outcomes (e.g., HIV or STI risk, adverse outcomes in pregnancy, and mucosal inflammation).^11^ Although women with CST-IV are much more likely to have BV, as many as 50% do not have BV.^11,12^ Investigators call for studies characterizing which women among those with nonoptimal vaginal CST-IV/molecular BV have clinical BV, because this may be beneficial in understanding and typifying pathologic states of nonoptimal vaginal microbiome. The objective of this study was to identify demographic and behavioral factors, symptoms, clinical findings, and genital microbiome composition in women and their male sex partners that differentiates women with and without clinical BV among those with nonoptimal CST-IV.

## METHODS

This study was approved by the ethical review committee of Maseno University (Kisumu, Kenya) and the institutional review board of the University of Illinois at Chicago.

### Study Design and Participants

Subjects in this analysis were enrolled in *Afya Jozi, Afya Jamii* (Kiswahili for “Healthy Pair, Healthy Community”), a prospective cohort study of heterosexual couples in Kisumu, Kenya. Recruitment and eligibility criteria have been published.^13^ Eligible members of couples independently confirmed they had been in a sexual relationship for at least 6 months duration and agreed to attend all study visits together. Eligible men were aged 18 to 35 years, and their female partners were 16 years and older. Couples in which one or both members had taken antibiotics within the past 30 days were not enrolled until 30 days had passed since completion of antibiotics. Research clinicians obtained written informed consent and conducted study procedures, including interviews, in the participant’s preferred language (English, Dho Luo, Kiswahili). Each member of the couple received 400 Kenyan shillings (~$4 US dollars) at each completed study visit. Couples were scheduled for follow-up at 1, 6, and 12 months after baseline. Couples were enrolled from April 1, 2014, to June 22, 2016, and 12-month follow-up was completed June 21, 2017.

### Data and Specimen Collection

At each visit, participants underwent a standardized medical history and physical examination and personal interview to obtain sociodemographic information and information on sexual behavior; men and women underwent study procedures separately in private examination rooms. At baseline and each follow-up visit, penile meatal swabs and cervicovaginal lavage were obtained for microbiome characterization. To obtain the meatal swab, the clinician applied light pressure and twirled a premoistened minitip flocked swab (Copan Diagnostics, Inc., Corona, CA) at the meatal depression for 3 to 5 rotations (the swab was not inserted into the urethra). Cervicovaginal lavage specimens were immediately aliquoted to 2.5-mL cryovials and, along with penile swabs, stored at −80°C until shipment. Before cervicovaginal lavage collection, the clinician collected 3 vaginal swabs for assessment of BV, whiff test, and wet mount microscopy. Slides for BV and wet mount microscopy were taken immediately to the on-site laboratory. If trichomonads were observed, this was recorded in the laboratory findings. After Gram staining, BV was evaluated according to Nugent’s criteria, in which a score of 7 to 10 is defined as BV.^8^ Treatment of BV was provided at point of care based on Amsel’s criteria^7^; women were treated with either 2 g oral tinidazole for 2 days or 400 mg oral metronidazole for 7 days.^9^ HIV (parallel rapid assays) and herpes simplex virus (HSV) type 2 (HSV-2 IgG ELISA, Kalon Biological Limited, Aldershot, United Kingdom) were measured at baseline and at 6 and 12 months, as previously detailed.^13^

### Vaginal and Penile Microbiome Characterization

DNA extraction was performed using EZ1 instrument, implementing the EZ1 DNA tissue protocol (Qiagen, Hilden, Germany). gDNA was used as template for PCR amplification of the V3–V4 variable region of bacterial 16S rRNA genes using a 2-stage PCR protocol with primers CS1_341F and CS2_806R.^14^ After pooling, amplicons were sequenced on an Illumina MiSeq instrument, implementing V3 chemistry (600 cycles). DNA extraction, library preparation, and sequencing were performed at the UIC Sequencing Core. Quality control and taxonomic annotation were conducted by the University of Maryland Institute for Genomic Science following previously a published protocol.^15^ Meatal microbiome data were filtered to retain taxa that contributed at least 0.01% of the total sequence reads. This resulted in selection of 57 taxa. Raw sequence data files are available in the Sequence Read Archive (National Center for Biotechnology Information; BioProject identifier PRJNA516684).

### Definition of Analytic Sample

Among 252 women, there were 732 observations at which both the vaginal microbiome and BV were measured. For analysis, we defined clinical BV according to Nugent’s criteria because of superior reproducibility and accuracy over Amsel’s criteria.^16,17^ Each vaginal microbiome observation was assigned to a CST based on its distance from the centroid of CSTs defined in a reference database of more than 13,000 vaginal communities characterized by 16s rRNA sequencing.^18^ This analysis was carried out by the Institute for Genomic Medicine at the University of Maryland, and methods are described by Brown et al.^19^ Overall, nonoptimal vaginal microbiome/molecular BV was present at 43.9% of the 732 observations: CST-IVA (n = 46) and CST-IVB (n = 275). Inferential analyses are restricted to these 321 CST-IV observations, as the goal of this analysis was to identify factors associated with clinical BV among women with nonoptimal vaginal microbiome/molecular BV. Penile microbiome measure was available for 307 of the 321 observations with nonoptimal vaginal microbiome.

### Statistical Analysis

#### Descriptive Analyses

We visualized differences in community composition by vaginal CST and Nugent BV status through heatmaps. For the penile microbiome, stacked bar plots summarize the relative abundance of the top 10 most common taxa (accounting for 68.2% of sequence reads), stratified by male circumcision status and female partner BV status. Among observations with nonoptimal CST-IV, analysis of similarity (ANOSIM) was used for global test of significance for comparison of bacterial communities by Nugent BV status. These analyses were conducted in Primer 7.0.^20^ We compared clinical characteristics and other laboratory findings by Nugent BV status but did not enter these variables in statistical modeling, because these features are more likely to be a result of or representative of BV rather than explanatory to BV. Similarly, alpha diversity measures (i.e., measures of bacterial community variation) are presented descriptively (*vegan* package, implemented in R^21^).

#### Inferential Analyses

To estimate the odds ratio of Nugent BV among women with nonoptimal CST/molecular BV, we applied the generalized estimating equation analysis, which incorporated the within-subject correlation among repeated measures, assuming binomial distributions with logit link. We compared demographic (age, educational attainment), behavioral (e.g., days since last sex, multiple sex partners, and condom use), and health factors (e.g., HIV status, HSV-2 serostatus, and contraceptive use) of women and male sex partners by BV status. All explanatory variables, except for age, educational attainment, and previous pregnancy, were assessed as time-varying covariates. Baseline values of HIV, HSV-2, number of sex partners in the past 6 months, and contraceptive use were carried forward to the 1-month observation and were time updated with the 6- and 12-month response values. Because male sex partner circumcision status and penile microbiome are associated with BV,^22,23^ we examined the association of Nugent BV with circumcision status and penile microbiome composition. To identify meatal taxa associated with Nugent BV, we first applied stability selection for feature selection (*stabs* package, implemented in R^24^). In this approach, we applied Lasso regression to 250 randomly generated subsets of the penile microbiome data and used a cutoff of *P* < 0.20 in combination with detection of a specific taxon in at least 50% of subsets. Lasso regression is a machine-learning algorithm used to identify taxa that relate to the target variable (BV). Stability selection strengthens our confidence in the selected taxa and reduces the likelihood of false-positive selections by identifying which taxa are important in a majority of sampled versions of the data; that is, the selected taxa have stable importance. Before feature selection, data were center log ratio transformed after geometric Bayesian multiplicative prior imputation of zeros (*zCompositions* package, implemented in R^25^), to address sparsity and while maintaining the same total number of reads.^26^

We first built a covariates-only model (model 1) and then a second model incorporating penile taxa (model 2) to enable direct interpretation of how covariates may change in the presence of penile taxa. For each model, variables statistically significant at the *P* < 0.20 level in univariate analyses were entered into multivariable analyses,^27^ with *P* < 0.10 for retention of variables in the multivariable model, after stepwise backward variable selection. Standard errors were obtained using an exchangeable correlation structure with robust estimation. Time was treated as a categorical variable. Sensitivity analyses excluding observations with intermediate Nugent score (4–6) are presented in supplemental files, http://links.lww.com/OLQ/A556. Generalized estimating equation modeling was conducted using Stata/SE 15.2 for Windows (Stata Corp., College Station, TX).

## RESULTS

**Figure 1:**
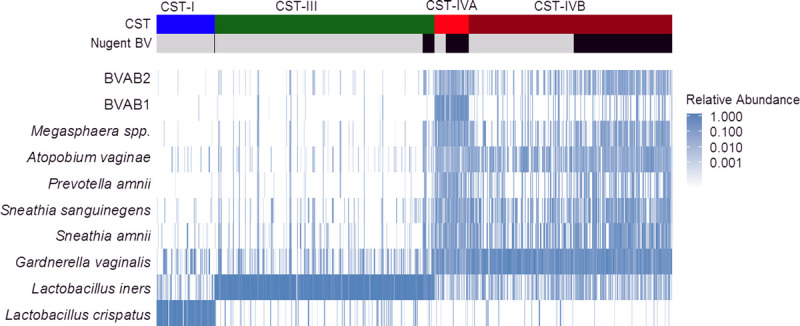
Heatmap summarizing relative abundance for the 10 most abundant vaginal taxa by CST and Nugent BV status. The heatmap represents the relative abundance of the 10 taxa with highest mean relative abundance, shown for each observation, clustered by CST and Nugent BV status. Greater relative abundance is shaded with increasing intensity of blue color. Observations with Nugent BV (Nugent score 7–10) are indicated with black coloring.

Among 732 observations (Table S1, Supplemental Digital Content, http://links.lww.com/OLQ/A556), we identified 6 CSTs: CST-I (*L. crispatus* dominant, n = 79 [10.8% of observations]), CST-II (*L. gasseri* dominant, n = 7 [0.96% of observations]), CST-III (*L. iners* dominant, n = 297 [40.6% of observations]), CST-IVA (*BVAB* dominant, n = 46 [6.3% of observations]), CST-IVB (*Gardnerella vaginalis* dominant, n = 275 [37.6% of observations]), CST-IVC (*Sneathia amnii* dominant, n = 19 [2.6% of observations]), and CST-V (*L. jensenii* dominant, n = 9 [1.2% of observations]). Figure 1 is a heatmap representing relative abundance of the most abundant taxa by CST and BV status; CST-II, CST-IVC, and CST-V are excluded because of sparsity and difficulty visualizing. Overall, Nugent BV was detected in 184 observations, 89% of which occurred in CST-IVA and CST-IVB (Table S1, Supplemental Digital Content, http://links.lww.com/OLQ/A556).

### Nonoptimal Vaginal CST Composition Varied by Nugent BV Status

**Figure 2:**
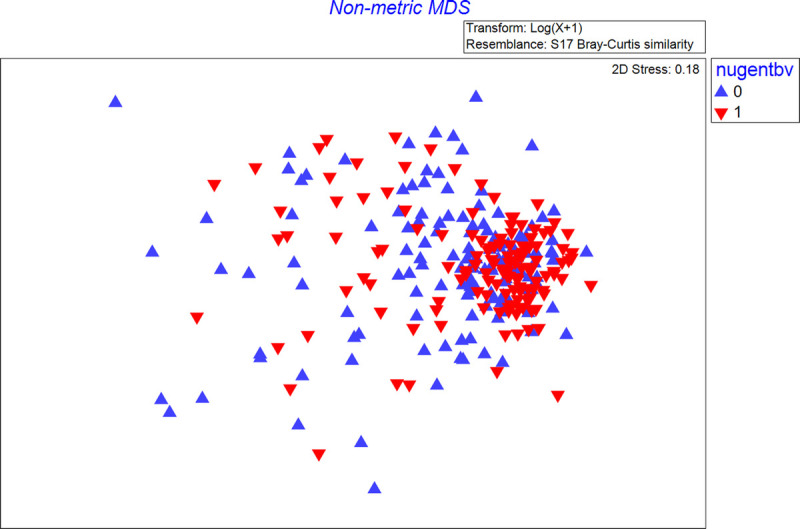
Nonmetric multidimensional scaling plot showing the similarity of vaginal microbiome community of women with and without BV (Nugent score 7–10 vs. 0–6), within CST IVB. The nonmetric multidimensional scaling plot (MDS) represents the pairwise Bray-Curtis dissimilarity between observations. Red triangles represent observations in which Nugent BV is detected (Nugent score 7–10), and blue triangles represent observations in which Nugent BV is absent (Nugent score 0–6).

Community state type IVA (relative abundance) comprised Clostridiales BV-associated bacteria-1 (BVAB*-*1; 20.5%), *G. vaginalis* (12.8%), *S. amnii* (9.6%), and *Sneathia sanguinegens* (7.9%), whereas CST-IVB comprised *G. vaginalis* (30.7%), *S. amnii* (11.7%), and *S. sanguinegens* (9.1%). Within CST-IVB, there were substantial differences in several taxa (ANOSIM, *P* = 0.001). *S. amnii, S. sanguinegens*, *Megasphaera* species, BVAB-2, and *Prevotella amnii* were enriched for observations with BV compared with observations without BV; these 5 taxa contributed to 17% of the overall differences in community composition by women’s BV status within CST-IVB (Table 1, Fig. 2). Among numerous other differential taxa, enriched taxa included *Prevotella* species (i.e., *Prevotella* identified at the genus level, but species was not identified) and several other *Prevotella* identified at the species level, along with *L. iners*, *Atopobium vaginae*, and species of *Dialister.* Alpha diversity indices (Shannon, Simpson, Richness, Evenness) were greater for observations in CST-IVA than in CST-IVB, and those within CST-IVB were greater for observations with BV than without (Table 2). Within CST-IVA, the microbiome composition did not differ by Nugent BV status (ANOSIM, *P* = 0.120; Supplementary Table S2, http://links.lww.com/OLQ/A556; Supplementary Figure S1, http://links.lww.com/OLQ/A557).

**TABLE 1:** Results of Similarity of Percentages Analysis: Taxa Contributing to 70% Dissimilarity Between Women With and Without Bacterial Vaginosis (BV) Within CST-IVB

Taxon ID: Taxa	Ave. Abundance*		Ave. Diss	Diss/SD	Contrib%	Cum.%
	BV Negative	BV Positive				
Species: *Sneathia amnii*	4.32	6.24	1.92	1.17	3.49	3.49
Species: *Sneathia sanguinegens*	4.28	5.90	1.87	1.19	3.40	6.89
Genus: *Megasphaera*	2.40	5.00	1.85	1.29	3.36	10.25
Species: BVAB-2	2.28	5.07	1.82	1.30	3.31	13.55
Species: *Prevotella amnii*	2.15	3.98	1.71	1.11	3.10	16.65
Species: *Prevotella timonensis*	3.47	5.00	1.57	1.24	2.86	19.51
Genus: *Prevotella*	2.04	4.24	1.56	1.27	2.84	22.35
Species: *Dialister succinatiphilus*	2.39	4.64	1.50	1.31	2.73	25.07
Species: *Lactobacillus iners*	3.91	4.30	1.50	1.28	2.71	27.79
Species: *Prevotella bivia*	2.56	2.26	1.42	1.01	2.58	30.37
Species: *Atopobium vaginae*	4.96	6.06	1.37	0.98	2.49	32.86
Genus: *Anaerococcus*	3.26	3.12	1.35	1.19	2.45	35.32
Species: *Aerococcus christensenii*	3.14	3.06	1.28	1.24	2.33	37.65
Family: Coriobacteriaceae	1.65	3.55	1.26	1.33	2.28	39.93
Genus: *Firmicutes* bacterium, unclassified	2.37	2.13	1.24	1.08	2.25	42.18
Species: *Gemella haemolysans*	2.23	2.73	1.21	1.18	2.19	44.36
Species: *Ralstonia pickettii*	2.83	1.89	1.18	1.17	2.15	46.51
Species: *Peptoniphilus gorbachii*	2.56	3.46	1.17	1.24	2.13	48.64
Family: Ruminococcaceae	1.04	2.78	1.16	1.20	2.11	50.75
Species: *Sediminibacterium salmoneum*	2.64	1.35	1.15	1.10	2.08	52.83
Species: *Finegoldia magna*	3.88	4.89	1.14	1.12	2.07	54.90
Species: *Ureaplasma urealyticum*	2.17	0.87	1.06	0.93	1.93	56.83
Species: *Peptoniphilus lacrimalis*	1.44	2.21	1.00	1.10	1.82	58.65
Species: *Dialister micraerophilus*	2.92	3.24	0.99	1.14	1.80	60.45
Species: *Mycoplasma hominis*	1.70	1.72	0.98	0.99	1.79	62.23
Species: *Porphyromonas asaccharolytica*	1.49	1.81	0.96	0.98	1.74	63.98
Species: *Peptostreptococcus anaerobius*	1.61	1.58	0.95	0.96	1.73	65.71
Family: Veillonellaceae	0.56	1.86	0.91	0.75	1.65	67.36
Species: BVAB*-*1	0.83	1.70	0.91	0.72	1.65	69.02
Species: *Prevotella disiens*	1.17	1.67	0.89	0.89	1.62	70.63

*Average abundance as presented in the table is natural log-transformed sequence counts averaged across subjects.

Ave Diss indicates average Bray-Curtis dissimilarity; Contrib%, percent contribution to dissimilarity between BV-negative and BV-positive communities; Cum.%, cumulative percent contribution to dissimilarity between BV-negative and BV-positive communities; Diss/SD, dissimilarity divided by SD.

**TABLE 2:** Distribution of Clinical and Laboratory Factors by Nugent BV Status Among Observations in Which the Vaginal Microbiome Is CST-IVA and CST-IVB

	CST-IVA (n = 46)		CST-IVB (n = 275)	
	No BV (n = 15), n (%)	BV (n = 31), n (%)	No BV (n = 142), n (%)	BV (n = 133), n (%)
Reported vaginal discharge	2 (13)	9 (29)	19 (3)	24 (18)
Clinician-detected vaginal discharge	8 (53)	19 (61)	31 (22)	75 (56)
Vaginal pH >4.5	8 (53)	22 (71)	59 (42)	106 (80)
Clue cells detected on wet mount	9 (60)	31 (100)	43 (30)	129 (97)
Whiff test positive	8 (53)	23 (74)	32 (23)	102 (77)
BV by Amsel’s criteria (≥3 criteria met)	8 (53)	23 (74)	23 (16)	100 (76)
Documented antimicrobial treatment of BV	9 (60)	27 (87)	53 (37)	120 (90)
Nugent score				
0–3	5 (33)		89 (63)	
4–6	10 (67)		53 (37)	
7–10		31 (100)		133 (100)
Alpha diversity measures, median (IQR)				
Shannon	2.35 (1.68–2.59)	2.34 (2.08–2.54)	1.66 (1.21–2.07)	2.05 (1.71–2.27)
Simpson	0.87 (0.75–0.89)	0.86 (0.80–0.89)	0.73 (0.57–0.81)	0.80 (0.72–0.84)
Richness	26 (23–37)	28 (25–33)	22 (15–28)	26 (22–31)
Evenness	0.69 (0.57–0.73)	0.70 (0.63–0.75)	0.55 (0.44–0.63)	0.63 (0.55–0.68)

*P* values are not estimated because the purpose of this analysis is descriptive rather than inferential and because the sample size (n) represents observations rather than individuals, and thus, there are repeated observations within individual.

IQR indicates Interquartile range.

Both CST-IVA and CST-IVB were relatively stable over time. Among women with any CST-IVA or CST-IVB observation at any time point, CST-III was the most likely alternative state, but the majority remained in CST-IVA or CST-IVB (Supplementary Table S3, http://links.lww.com/OLQ/A556; Supplementary Figure S2, http://links.lww.com/OLQ/A558). Among 97 women with CST-IVB at baseline, 44.3% remained free of BV throughout observation, 24.7% had one observation of BV, and 30.9% had 2 or more observations with BV. Among 14 women with CST-IVA at baseline, only 3 (21%) remained free of BV throughout follow-up, whereas 4 (28.6%) had 1 observation of BV and 7 (50%) had BV at 2 or more observations.

### The Penile Microbiome Differed by Men’s Circumcision Status and Female Partner BV Status

**Figure 3:**
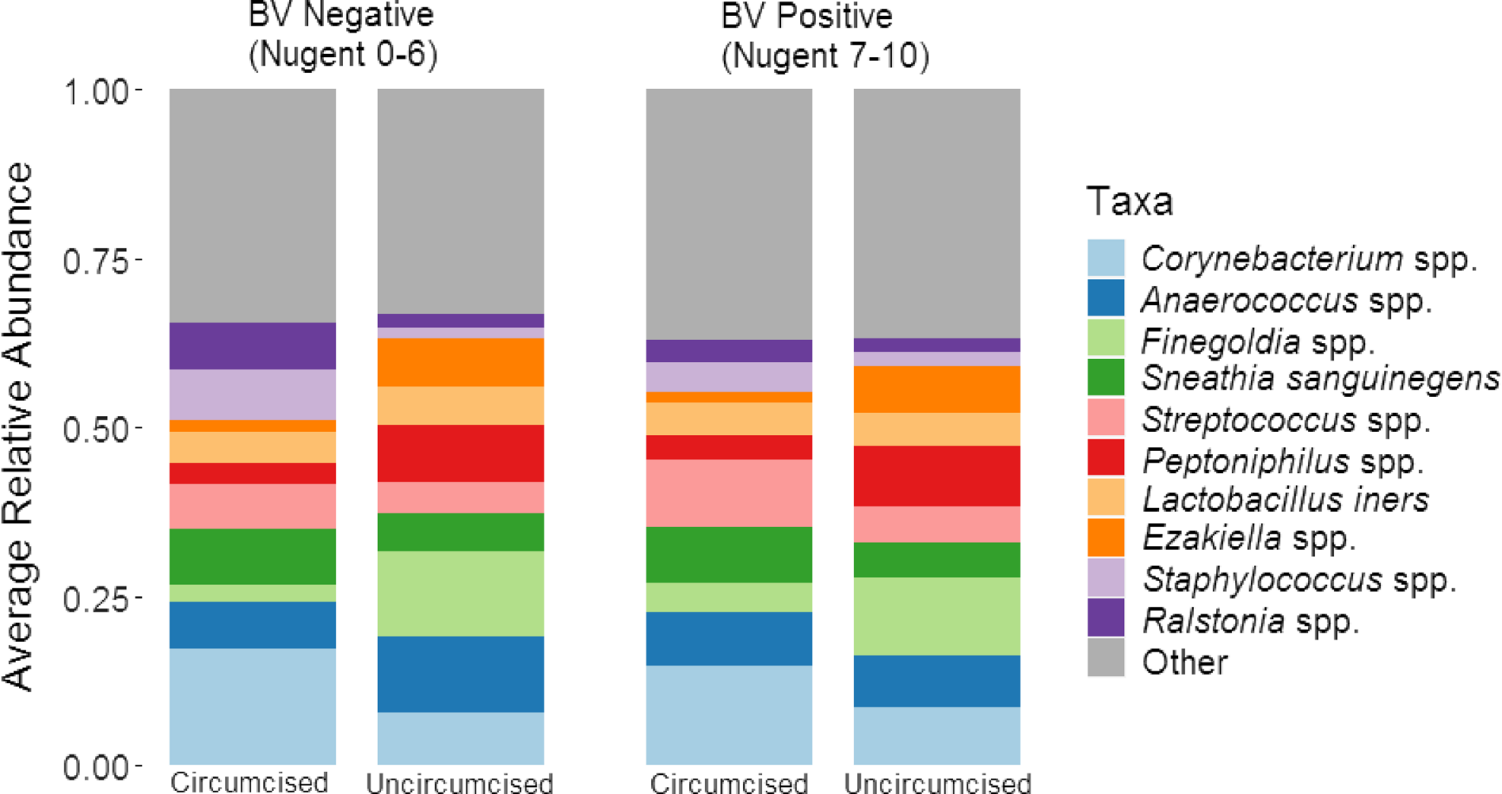
Stacked bar charts summarizing the mean relative abundance of 10 meatal taxa with the highest relative abundance by circumcision status and female partner BV status. These stacked bar charts summarize the mean relative abundance (*y* axis) of 10 penile taxa with the highest mean relative abundance, stratified by Nugent BV status and man’s circumcision status.

Ten penile taxa with the highest relative abundance accounted for 68% of all sequence reads (Table 3). The penile taxa with the highest relative abundance were *Corynebacterium* (12.6%), *Anaerococcus* (8.3%), *Finegoldia* (7.3%), *S. sanguinegens* (6.9%), and *Streptococcus* (6.8%) and differed substantially by men’s circumcision status (Fig. 3). Stability selection resulted in selection of 7 penile taxa (fraction of times selected): *Dialister* (0.54)*, Aerococcus* (0.56)*, Fastidiosipila* (0.62), *Fusobacterium* (0.65), *Brevibacterium* (0.76), *Ureaplasma* (0.87), and *Megasphaera* (0.97; Table 3). The presence and relative abundance of the taxa identified by stability selection had minor absolute differences in relative abundance, although some had 2- to 3-fold relative differences (Table 3).

**TABLE 3:** Presence and Mean Relative Abundance by Circumcision and BV Status for the 10 Most Abundant Meatal Taxa and Those Identified by Stability Selection

	Circumcised		Uncircumcised	
	BV Negative (Nugent 0–6; n = 92), % Present (% Mean RA)	BV Positive (Nugent 7–10; n = 74), % Present (% Mean RA)	BV Negative (Nugent 0–6; n = 63), % Present (% Mean RA)	BV Positive (Nugent 7–10; n = 78), % Present (% Mean RA)
Most abundant taxa				
*Corynebacterium*	98.9 (17.9)	100 (15.8)	93.7 (8.74)	91.0 (9.60)
*Anaerococcus*	95.7 (7.51)	96.0 (8.49)	100 (11.7)	97.4 (8.00)
*Streptococcus*	83.7 (8.05)	83.8 (12.4)	74.6 (6.29)	69.2 (7.92)
*L. iners*	47.8 (10.2)	41.9 (11.7)	50.8 (11.1)	50.0 (10.6)
*Staphylococcus*	95.7 (7.87)	98.7 (4.60)	71.4 (2.14)	76.9 (2.87)
*Finegoldia*	91.3 (3.09)	93.2 (4.96)	100 (12.7)	96.2 (11.8)
*S. sanguinegens*	56.5 (14.3)	59.5 (14.1)	55.6 (10.4)	60.3 (8.54)
*Peptoniphilus*	84.5 (3.66)	93.2 (3.95)	100 (9.03)	93.6 (9.62)
*Ezakiella*	75.0 (2.50)	71.6 (2.46)	85.7 (8.91)	85.9 (8.25)
*Ralstonia*	54.4 (13.9)	68.9 (5.29)	73.0 (3.24)	70.5 (3.22)
Taxa identified by stability selection				
*Dialister*	50.0 (0.63)	66.2 (0.92)	84.1 (1.25)	80.8 (1.58)
*Aerococcus*	54.4 (0.61)	50.0 (0.82)	52.4 (0.33)	44.9 (0.31)
*Fastidiosipila*	47.0 (1.10)	55.4 (0.55)	47.6 (0.41)	56.4 (0.79)
*Fusobacterium*	33.7 (1.70)	36.5 (3.79)	27.0 (2.02)	21.8 (1.23)
*Brevibacterium*	62.0 (0.27)	71.6 (0.58)	34.9 (0.22)	46.2 (0.46)
*Ureaplasma*	66.3 (3.46)	68.9 (1.03)	61.9 (0.92)	50.0 (1.27)
*Megasphaera*	10.9 (0.86)	31.1 (0.36)	15.9 (0.57)	30.8 (0.38)

*n = 307; penile microbiome measure missing for 14 observations among the 321 observations with nonoptimal vaginal microbiome.

% mean RA indicates the percent mean relative abundance of the specified taxa in the observations in which it is present; % present, the proportion of observations in which there is any amount of the specified taxa.

### Within Nonoptimal CST, Clinical and Laboratory Features Differed by Nugent BV Status

Overall, only 16.8% of women with CST-IV reported vaginal discharge, although 41.4% of women had vaginal discharge detected on examination (Table 2). A greater proportion of observations with Nugent BV had clinician-detected vaginal discharge, elevated vaginal pH (>4.5), whiff test–positive samples, and clue cells detected on wet mount. Among observations in which BV was not diagnosed, a greater proportion of observations had a Nugent score of 0 to 3 for CST-IVB (63%) than for CST-IVA (33%), although observations for CST-IVA without BV were infrequent. For observations in which BV was not diagnosed, compared with CST-IVA, observations classified as CST-IVB were less likely to have reported vaginal discharge, clinician-detected vaginal discharge, elevated vaginal pH, detection of clue cells, and positive whiff test (and accordingly, BV by Amsel’s criteria). Alpha diversity measures were greater for observations in CST-IVA than in CST-IVB, similar by BV status within CST-IVA, and modestly higher among women with BV within CST-IVB.

### Within Nonoptimal CST, Women With CST-IVA, HIV, and HSV-2 Were More Likely to Have Nugent BV

At baseline, women were a median age of 22 years, 16% had HIV infection, and 66% had HSV-2 seropositive (Table 4). The median age of male sex partners was 27 years, 12% were HIV positive, 48% HSV-2 were seropositive, and 55% were circumcised. In univariate analysis, women with Nugent BV were more likely to be in CST-IVA and more likely to be HIV positive, HSV-2 seropositive, and with *Trichomonas vaginalis* detected on wet mount (Table 4). The male sex partners of women with Nugent BV were less likely to be circumcised, and more likely to be HIV positive and to report using condoms at the last sexual intercourse. In multivariable adjusted analyses, the odds of BV were increased for women with CST-IVA (vs. CST-IVB; adjusted odds ratio [aOR], 1.90; 95% confidence interval [CI], 0.97–3.73; *P* = 0.063), HSV-2 (aOR, 1.62; 95% CI, 0.92–2.85; *P* = 0.095), or HIV (aOR, 2.48; 95% CI, 1.16–5.34; *P* = 0.020; Table 5, model 1). In a sensitivity analysis excluding observations with intermediate Nugent score (4–6), the associations of women’s covariates (CST, HSV-2, and HIV status) remained of similar magnitude, although the protective association of circumcision status was strengthened and statistically significant (aOR, 0.45; 95% CI, 0.24–0.84; Supplementary Table S4, model 1, http://links.lww.com/OLQ/A556).

**TABLE 4:** Distribution of Demographic, Behavioral, and Health Status Factors by Nugent’s BV Status at Baseline and Follow-Up Visits, and Results of Time-Adjusted Univariate Generalized Estimating Equation Analysis

	Baseline		1 mo		6 mo		12 mo		Time-Adjusted Univariate Odds Ratio (95% CI)
Study Visit	No BV (n = 59), n (%)	BV (n = 47), n (%)	No BV (n = 38), n (%)	BV (n = 39), n (%)	No BV(n = 30), n (%)	BV (n = 42), n (%)	No BV (n = 30), n (%)	BV (n = 36), n (%)	
Community state type									
IV-A	5 (9)	8 (17)	4 (11)	4 (10)	3 (10)	12 (29)	3 (10)	7 (19)	1.90 (1.07–3.35)*
IV-B	54 (91)	39 (83)	34 (89)	35 (90)	27 (90)	30 (71)	27 (90)	29 (81)	Reference
Demographic and behavioral factors									
Women									
Age, mean (IQR; fixed), y	22 (20–25)	22 (20–25)	21 (20–27)	23 (21–26)	21 (20–23)	24 (21–26)	23.5 (21–28)	22.5 (21–26)	1.02 (0.96–1.08)
Educational attainment (fixed)									
Primary or less	27 (46)	25 (53)	17 (45)	23 (59)	19 (63)	24 (57)	15 (50)	17 (47)	Reference
Some secondary, secondary	25 (42)	17 (36)	14 (37)	13 (33)	8 (27)	13 (31)	9 (30)	14 (39)	0.94 (0.53–1.67)
Postsecondary	7 (12)	5 (11)	7 (18)	3 (8)	3 (10)	5 (12)	6 (20)	5 (14)	0.65 (0.30–1.40)
≥2 sex partners past 6 mo	1 (1.8)	2 (4.4)	1 (3.1)	2 (5.9)	0 (0)	1 (2.4)	1 (3.7)	2 (5.7)	1.86 (0.46–7.49)
Days since last sexual intercourse									
1–2	24 (41)	24 (51)	16 (42)	17 (44)	12 (40)	10 (24)	12 (40)	9 (25)	Reference
3–6	18 (31)	15 (32)	6 (16)	9 (23)	10 (33)	14 (33)	5 (17)	7 (19)	1.15 (0.70–1.90)
≥7	17 (29)	8 (17)	16 (42)	13 (33)	8 (27)	18 (43)	13 (43)	20 (56)	1.12 (0.66–1.90)
How soon clean vagina after sex (<1 vs. ≥1 h)	35 (59)	34 (72)	23 (68)	24 (67)	21 (70)	28 (67)	23 (77)	28 (78)	0.85 (0.51–1.42)
Men									
Age, median (IQR), y	26 (24–30)	27 (23–30)	27 (24–31)	27 (23–31)	26 (24–30)	27 (24–32)	27 (25–30)	27 (24–32)	1.00 (0.94–1.06)
Educational attainment (fixed)									
Primary or less	24 (41)	19 (40)	17 (45)	15 (38)	13 (43)	20 (48)	12 (40)	12 (33)	Reference
Some secondary, secondary	24 (41)	23 (49)	16 (42)	19 (49)	11 (37)	17 (40)	12 (40)	18 (50)	1.19 (0.68–2.09)
Postsecondary	11 (19)	5 (11)	5 (13)	5 (13)	6 (20)	5 (12)	6 (20)	6 (17)	0.70 (0.32–1.52)
≥2 sex partners past 6 mo	18 (31)	15 (33)	13 (34)	13 (34)	4 (13)	6 (16)	5 (19)	9 (32)	1.13 (0.65–1.96)
Condom used at last sexual intercourse	5 (8.5)	9 (19)	5 (13.5)	7 (18)	3 (10)	8 (21)	4 (14)	6 (21)	1.85 (0.98–3.49)^†^
Clinical factors									
Women									
HSV-2 seropositive	35 (59)	35 (74)	20 (53)	31 (79)	19 (63)	28 (67)	23 (77)	24 (67)	1.63 (0.94–2.83)^†^
HIV positive	6 (10)	11 (23)	3 (8.1)	7 (18)	1 (3.3)	11 (27)	5 (17)	7 (20)	2.47 (1.21–5.03)*
*Trichomonas vaginalis* detected on wet mount	4 (6.8)	2 (4.3)	1 (2.6)	1 (2.6)	0 (0)	6 (14)	1 (3.3)	2 (5.6)	2.41 (0.92–6.32)^†^
Previous pregnancy (fixed)	50 (85)	40 (85)	32 (84)	33 (85)	25 (83)	39 (93)	28 (93)	31 (86)	1.02 (0.53–1.95)
Contraception and pregnancy									
Oral contraceptive use	0 (0)	3 (6.4)	2 (5.9)	0 (0)	0 (0)	0 (0)	2 (6.7)	1 (2.8)	Reference
Injection contraceptive use	12 (20)	2 (4.3)	3 (8.8)	4 (11)	6 (21)	7 (17)	5 (17)	7 (19)	0.67 (0.17–2.67)
Implant contraceptive use	14 (24)	12 (26)	9 (27)	8 (22)	11 (38)	15 (37)	11 (37)	9 (25)	0.83 (0.21–3.22)
None, condoms, IUD, other^‡^	28 (47)	27 (57)	17 (50)	23 (64)	8 (28)	18 (44)	12 (40)	5 (42)	1.23 (0.34–4.47)
Pregnant (urine HCG positive)	5 (8.5)	3 (6.4)	3 (8.8)	1 (2.8)	4 (14)	1 (2.4)	0 (0)	4 (11)	0.70 (0.15–3.38)
Men									
HSV-2 seropositive	28 (48)	22 (47)	17 (46)	19 (49)	18 (60)	21 (54)	18 (62)	18 (62)	0.94 (0.57–1.55)
HIV positive	5 (8.8)	7 (15)	3 (8.3)	8 (21)	2 (6.7)	7 (18)	3 (10)	6 (21)	2.18 (0.99–4.80)^†^
Circumcised	34 (58)	24 (51)	25 (68)	19 (49)	18 (60)	15 (41)	15 (52)	15 (52)	0.66 (0.39–1.10)

“Fixed” refers to variables that do not change after baseline.

*P < 0.05.

^†^0.05 < *P* < 0.10.

^‡^This category of contraception includes 39 observations with no contraception, 2 observations with IUD, 11 observations with condoms as main method of contraception, 1 observation of lactation amenorrhea, 1 observation of tubal ligation, and 1 observation emergency pills.

**TABLE 5:** Results of Crude and Multivariable-Adjusted Generalized Estimating Equation Analysis: Factors Associated With Nugent’s BV Among Women With Community State Types CST-IVA or CST-IVB

	Time-Adjusted Univariate, Odds Ratio (95% CI), *P* Value	Multivariable-Adjusted Model 1* Covariates Only (n = 303), Adjusted Odds Ratio (95% CI), *P* Value	Multivariable-Adjusted Model 2* Covariates and Meatal Taxa (n = 296), Adjusted Odds Ratio (95% CI), *P* Value
CST-IVA vs. CST-IVB	1.90 (1.07–3.35), *P* = 0.027	1.90 (0.97–3.73), *P* = 0.063	1.91 (0.91–4.00), *P* = 0.087
Woman is HSV-2 seropositive	1.63 (0.94–2.83), *P* = 0.083	1.62 (0.92–2.85), *P* = 0.095	1.75 (0.97–3.17), *P* = 0.065
Woman is HIV positive	2.47 (1.21–5.03), *P* = 0.013	2.48 (1.16–5.34), *P* = 0.020	2.30 (1.00–5.32), *P* = 0.051
*T. vaginalis* detected on wet mount	2.41 (0.92–6.32), *P* = 0.074		
Male partner is HIV positive	2.18 (0.99–4.80), *P* = 0.052		
Male partner is circumcised	0.66 (0.39–1.10), *P* = 0.109	0.73 (0.43–1.24), *P* = 0.243	0.79 (0.45–1.41), *P* = 0.427
Male partner reported using condom at last sex	1.85 (0.98–3.49), *P* = 0.058		
Penile relative abundance^†^: *Dialister* species			1.16 (1.01–1.34), *P* = 0.034
Penile relative abundance^†^: *Megasphaera* species			1.22 (1.09–1.37), *P* = 0.001
Penile relative abundance^†^: *Brevibacterium* species			1.13 (1.02–1.25), *P* = 0.019
Visit			
Baseline	Reference	Reference	Reference
1 mo	1.23 (0.77–1.96), *P* = 0.396	1.32 (0.79–2.21), *P* = 0.282	1.66 (0.96–2.88), *P* = 0.069
6 mo	1.63 (0.93–2.88), *P* = 0.089	1.36 (0.74–2.50), *P* = 0.322	1.49 (0.77–2.88), *P* = 0.231
12 mo	1.41 (0.80–2.48), *P* = 0.234	1.05 (0.55–1.98), *P* = 0.890	1.17 (0.60–2.28), *P* = 0.653

*Model 1 and model 2 are simultaneously adjusted for all variables presented under each.

^†^Penile relative abundance data are center log ratio transformed before analysis.

### Penile Bacteria Were Associated With Increased Odds of Nugent BV Among Women With Nonoptimal CST

In multivariable modeling including covariates from model 1, increasing penile relative abundances of *Dialister* species, *Megasphaera* species, and *Brevibacterium* species remained statistically significantly associated with increased odds of BV in the female partner (Table 5, model 2). Women with CST-IVA, HSV-2, and HIV had increased odds of BV with similar coefficients and CIs as in model 1. Controlling for penile taxa, male partner circumcision status was not associated with BV (aOR, 0.79; 95% CI, 0.45–1.41). In a sensitivity analysis excluding observations with intermediate Nugent score (4–6), the coefficients of female covariates and penile taxa remained similar, although the protective association of circumcision status was strengthened and remained statistically significant even when controlling for penile taxa (Supplementary Table S4, model 2, http://links.lww.com/OLQ/A556).

## DISCUSSION

### Main Findings

(1) Although 89% of Nugent BV cases occurred among women with nonoptimal vaginal CST-IV, 49% of CST-IV observations did not have Nugent BV. (2) Among women with nonoptimal CST-IV, the vaginal microbiome was not homogenous, with enrichment of *S. amnii, S. sanguinegens*, *Megasphaera*, BVAB*-2*, and *P. amnii* among women with BV. (3) Among women with nonoptimal CST-IV, Nugent BV was more likely for observations with CST-IVA, HIV, or HSV-2, and male sex partner enrichment of specific penile bacteria.

### Interpretation

(1) Our findings are in keeping with other studies that demonstrate a substantial proportion of women have persistently low or moderate relative abundance of vaginal lactobacilli and absence of BV; this is generally more common among African American and African women.^1^ It is possible that these bacterial communities are functioning in ways that replicate the protective mechanisms of lactobacilli (e.g., lactic acid production and preventing biofilm formation), or there may be host-mediated mechanisms that contribute to or prevent a pathological state in non–lactobacillus-dominant communities. For example, the amount of glycogen or α-amylase production or variation in Toll-like receptors could explain variability in Nugent BV among women with nonoptimal vaginal microbiome.^28,29^ If stable, low to moderate vaginal lactobacillus and diverse CST is a homeostatic state, then antimicrobial, live biotherapeutic, or other interventions to alter CST may have adverse effects.^30^ On the other hand, studies have demonstrated that women with CST-IV have increased mucosal inflammation and epithelial barrier disruption, and subsequent risk of HIV acquisition, regardless of BV status, symptoms, or other STIs,^31s–33s^

(2) Women with CST-IVA (BVAB-1 dominant) vaginal community were more likely to have Nugent BV and symptoms, signs, or microscopy findings associated with BV. A review by Marrazzo^34s^ summarizes mechanisms for increased risk of BV with several BVAB species: contribution to biofilm formation, potential antibiotic resistance, potential penile reservoir, and ability to establish dominance in the vaginal community. *G. vaginalis* is highly prevalent in women across many studies; Muzny and Schwebke^35s^ indicate *G. vaginalis* is likely copathogenic, requiring other bacteria or other factors to initiate the conversion to BV. These differences in pathogenesis may explain why women with the CST-IVA subtype had a greater likelihood of BV. Comprehensive modeling of temporal dynamics is outside the scope of the current analysis; however, for most women in our sample who *ever* had CST-IVA or CST-IVB, CST-IV was persistent, despite nearly all women with BV having documented treatment. Longitudinal studies evaluating host factors and bacterial function among women with persistent, nonoptimal CST may identify potential protective mechanisms against BV, and factors preceding shifts from nonoptimal CST without BV to nonoptimal CST with BV.

A meta-analysis demonstrates increased risk of BV among women with HSV-2^36s^; Esber et al.^37s^ suggest host response and HSV-2 viral expression lead to a vaginal environment that inhibits healthy vaginal flora. Our analysis shows the association between HSV-2 and BV holds true even *within* nonoptimal CST. Many HSV-2 viral genes encode for products that are toxic to epithelial cells, and HSV-2 engages CD8^+^ cell activity, which may contribute to perturbation of the vaginal microbiome during reactivations. Bacterial vaginosis increases the risk of HIV acquisition^3^ and transmission^38s^, and given the chronic, recurrent nature of BV, they are more likely to be codetected. Currently, guidelines do not recommend screening and treatment of BV in asymptomatic women. For women with HIV or HSV-2, screening and treatment of BV in the absence of symptoms may have benefits related to reduced frequency of HSV-2 outbreaks, mucosal inflammation, or HSV-2 or HIV shedding. We are unaware of published studies evaluating the potential utility of BV screening and treatment among asymptomatic women with HSV-2 and HIV in relation to these outcomes.

(3) As defined by Verstraehlen et al.,^39s^ BV is a “sexually enhanced” condition with substantial epidemiologic and microbiologic evidence demonstrating increased risk with increasing sexual exposures.^40^ Female sex partners of Ugandan men undergoing voluntary medical male circumcision had a 40% lower prevalence of BV at 1 year after circumcision.^22^ A subsequent study showed that BV among female partners was associated with greater penile enrichment of BV-associated anaerobic bacteria at the coronal sulcus^23^ among uncircumcised compared with circumcised men.^41s^ We found that enrichment of penile *Dialister* species, *Megasphaera* species, and *Brevibacterium* species was positively associated with BV (*Megasphaera* species and *Dialister* species were also enriched in the vaginal microbiomes of women with BV, although *Brevibacterium* was not). The protective effect of male partner circumcision on BV likely extends to women with nonoptimal vaginal communities, CST-IVA or CST-IVB. When we excluded from the analysis women with intermediate Nugent scores (Supplementary Table S4, http://links.lww.com/OLQ/A556), the protective effect of having a circumcised male partner was strengthened; this makes sense because women with intermediate Nugent scores may represent misclassified observations of BV or observations that are more likely to progress to BV. Alternatively, these bacteria may not be residents of the penile microbiome and instead may be mechanically transferred back and forth; thus, the penile microbiome serves as a reflective pool rather than a potential reservoir. However, BV-related bacteria are also found in men’s urine and semen. As reported by Nelson et al.,^42s^ the microbiota of men’s urine contained high abundances of bacteria that are also found in the vagina, and Mändar et al.^43s^ found a high concordance of microbiota between semen and vaginal samples, supporting their hypothesis that “semen serves as a medium for the transmission of microorganisms between men and women.” Given the high prevalence and high relative abundances of these BV-related bacteria recovered from sites throughout the male genitourinary system (semen, urethra, urine, glans, coronal sulcus) in our studies and those of others,^23s,41s–43s^ it is likely that microbiologic paradigms of the penis should consider the possibility that these bacteria reside there.

### Strengths and Limitations

Although there may have been misclassification, the distribution of BV by CST and subtype (Supplementary Table S1, http://links.lww.com/OLQ/A556) indicates that specificity was very high, with only 3 of 184 BV cases in the entire cohort occurring in CST-I or CST-II. Therefore, misclassification likely represents underestimation of BV, as suggested by our sensitivity analysis. Only 3 cases of Nugent BV were classified within 19 observations of CST-IVC, and we therefore excluded CST-IVC from analyses because of sparsity and inability to draw inference on the small number of cases in this CST. We did not measure other STIs such as chlamydia or gonorrhea, and these can also influence the composition of the vaginal microbiome.^44^ We did not measure bacterial load of any taxa, and this would have provided improved insights into the relation of penile microbiota to BV and symptoms in female partners, because absolute abundance more accurately represents changes in taxa (as it does not suffer the statistical constraint of compositionality).^45^ A limitation inherent in amplicon sequencing is annotation of bacteria. Although a standardized and replicable approach was applied, this algorithm has not been optimized for the penile microbiome. Several associations did not reach statistical significance at the *P* < 0.05 level. Although sample size affected precision of estimates, the magnitude of coefficients was stable across different modeling conditions, and results are in keeping with known findings of biological relevance. This study adds to the literature regarding epidemiologic and clinical understanding of molecular BV with several strengths: prospective and multiple sampling of cervicovaginal microbiota paired with male sex partner penile microbiota, measurement of HIV and HSV-2 status, standardized assessment of clinical symptoms and signs, and behavioral practices. An advantage of this study is that we recruited couples from the community rather than among women seeking clinical care related to BV. This more likely represents the average associations with BV in a setting of high HIV and HSV-2 prevalence. However, loss to follow-up may affect generalizability of results.

In this cohort of community-recruited Kenyan women with nonoptimal CST-IV/molecular BV, the vaginal microbiome composition was not homogeneous and was enriched with *S. amnii, S. sanguinegens, Megasphaera*, BVAB*-2*, and *P. amnii* among observations with clinical BV. For women with clinical BV, the penile microbiome of sex partners was enriched with taxa that are also associated with BV in women. Evaluation of the potential utility of BV screening among asymptomatic women with HIV and HSV-2 is warranted, given the increased rates of Nugent BV for these women. Interventions to modify the penile microbiome or to interrupt sexual exchange of BV associated bacteria within partnerships may reduce BV in female partners.

## Appendix

For further references, please see “Supplemental References,” http://links.lww.com/OLQ/A559.
